# Non-surgical Treatment May be Appropriate for Most Chinese Children With Monogenic Congenital Hyperinsulinism Based on a Retrospective Study of 121 Patients

**DOI:** 10.1155/2024/3961900

**Published:** 2024-11-19

**Authors:** Ming Cheng, Chang Su, Dongmei Wang, Yanning Song, Yang Li, He Zeng, Zheng Yuan, Xiaoqiao Li, Xi Meng, Yuan Ding, Bingyan Cao, Chunxiu Gong

**Affiliations:** ^1^Department of Endocrinology, Genetics, Metabolism, Beijing Children's Hospital, Capital Medical University, National Center for Children's Health 100045, Beijing, China; ^2^Beijing Key Laboratory for Genetics of Birth Defects, Beijing Children's Hospital, Capital Medical University, National Center for Children's Health 100045, Beijing, China; ^3^MOE Key Laboratory of Major Diseases in Children, Beijing Children's Hospital, Capital Medical University, National Center for Children's Health 100045, Beijing, China

**Keywords:** genotype-phenotype correlations, high-frequency variants, monogenic congenital hyperinsulinism, non-surgical treatment

## Abstract

**Objective:** There is a notable absence of extensive Chinese studies involving monogenic congenital hyperinsulinism (CHI). The purpose of this large retrospective Chinese cohort with monogenic CHI from a national children's medical center was to analyze the genetic and clinical characteristics.

**Methods:** We compared clinical characteristics grouped by genotypes based on CHI-targeted next-generation sequencing (tNGS) and performed subgroup analyses by onset time.

**Results:** Totally, 121 non-consanguineous patients were enrolled. Among them, 79 patients (65.3%) had variants in ATP-sensitive potassium channel (*KATP*) genes (62 heterozygotes and 17 compound heterozygotes), 35 (28.9%) in glutamate dehydrogenase 1 (*GLUD1*), and 7 (5.8%) in rare genes (hydroxyacyl-CoA dehydrogenase [*HADH*], glucokinase [*GCK*], and hepatocyte nuclear factor 4 alpha [*HNF4A*]). Ten patients had ATP binding cassette subfamily C member 8 (*ABCC8*) variants (p.G111R), and 12 had *GLUD1* variants (p.S498L), suggesting two potential founder variants. Three *ABCC8* variants (p.G1478R, p.L580_S581insFASL, and p.S986*⁣*^*∗*^) and two *HNF4A* variants (p.R63W and p.V382I) were previously reported to be associated with diabetes. Non-surgical treatment was effective in 65.9% of patients with *KATP* variants, while in 100% of those with non-*KATP* variants. For the subgroup of *KATP* variants, neonatal-onset patients tended to present with mild symptoms (67.9% versus 19.3%), had a higher proportion of surgical intervention (24.5% versus 3.8%), and displayed higher levels of serum insulin and C-peptide than non-neonatal onset ones (*p* < 0.001).

**Conclusion:** The absence of homozygous variants in *KATP* genes and a quite higher proportion of *GLUD1* variants than previous cohorts, may explain a high response rate of non-surgical treatment in this study. Surgery might be considered for neonatal-onset children, especially when *KATP* variants were discovered but not for those carried variants reported to cause diabetes in later life. While expanding the genotypic spectrum, we also highlight the clinical significance of genetic screening.

## 1. Introduction

Congenital hyperinsulinism (CHI) constitutes a heterogeneous disorder characterized by inappropriate secretion of insulin from pancreatic *β*-cells, resulting in severe hypoglycemia in neonates and infant [1]. The estimated incidence in live births ranges from 1 in 50,000 to 1 in 28,000 in the outbred populations [2, 3]. The clinical spectrum of CHI is diverse, with seizures or unconsciousness being the most severe symptoms, while nonspecific symptoms like hypothermia, lethargy, and hypotonia pose challenges in accurate identification [4].

To date, 16 crucial genes implicated in insulin secretion have been reported in association with CHI, such as ATP binding cassette subfamily C member 8 (*ABCC8*), potassium inwardly rectifying channel subfamily J member 11 (*KCNJ11*), glutamate dehydrogenase 1 (*GLUD1*), glucokinase (*GCK*), hydroxyacyl-CoA dehydrogenase (*HADH*), hepatocyte nuclear factor 1-alpha (*HNF1A*), hepatocyte nuclear factor 4 alpha (*HNF4A*), and solute carrier family 16 member 1 (*SLC16A1*) [5, 6]. These genes have been identified in 36%–69% of cases through genetic testing [7–10]. *ABCC8* variants predominantly account for the genetic basis, explaining 55%–100% of positive variant cases, while in *KCNJ11* and *GLUD1* display varying proportions [11–14]. However, there is a dearth of large-cohort studies in the Chinese population with only a few research endeavors providing a comprehensive overview of genotype–phenotype correlations in China [15–17].

Patients carrying *ABCC8*/*KCNJ11* (ATP-sensitive potassium channel [*KATP]*) variants exhibit early onset and refractory, although the association between symptom severity and pancreatic islet function remains unclear [18]. Biallelic recessive *KATP* variants manifest as diffuse CHI, whereas focal CHI typically results from inheriting paternal variants at 11p15.1 with the loss of maternal allele [19]. Given the high cure rate of surgery for focal CHI, surgical intervention is recommended in appreciable quantity of previous researches [19, 20]. Some evidence suggests conservative treatments can decrease the frequency of hypoglycemic episodes for diffuse CHI, with some patients experiencing spontaneous remission during long-term follow-up [21–23]. In addition, occurrence of postsurgery endocrine and exocrine dysfunction suggests a trend towards conservative therapy over surgery [19, 21]. Activating variants of *GLUD1* represent another common cause of monogenic CHI, with affected children typically being feeding-responsive and/or diazoxide-responsive. Unlike urea cycle defects, patients often display mild to moderate elevated serum ammonia without symptoms. Approximately one-third of affected children exhibit neurological disorders such as seizures and behavioral disturbances, even those maintained blood glucose well [24, 25].

In this current cohort, we investigated the clinical and genetic characteristics of 121 children with monogenic CHI over the past 16 years. Motivated by our previous research [26], the objective is to explore unique genotype–phenotype correlations within the Chinese population on the basis of an enlarged sample size.

## 2. Methods

### 2.1. Patients and Data Collection

This study included 121 cases of Chinese children with monogenic CHI treated at a national children's medical center, Beijing children's hospital between January 2006 to June 2023. Patients with any specific phenotypes associated with syndromes (e.g., dysmorphic features, hypopituitarism, and gastrointestinal abnormalities) have been excluded [27]. Clinical data encompassed gestational age, birth weight, onset time, and symptoms, insulin and C-peptide levels (when venous blood glucose <2.8 mmol/L), plasma ammonia levels (reference 2–72 μmmol/L), therapeutic options (including frequent feeding, diazoxide, octreotide, surgery, and quit treatment), and the dosages of diazoxide and octreotide.

The clinical diagnostic criteria for CHI adhered to current consensuses [28]. In neonatal cases, an increased intravenous glucose infusion rate (>8 mg/kg/min) was considered a supportive criteria. Macrosomia is defined as the neonatal birth weight ≥4000 g. To simplify statistical variables, seizure or unconsciousness assigned to severe symptoms, while asymptomatic hypoglycemia or lethargy to mild symptoms. Non-surgical treatment included dietary therapy and pharmacological intervention, such as diazoxide and octreotide.

Responsiveness to conservative treatment was defined as patients who successfully maintained blood glucose levels higher than 3.33 mmol/L without intravenous glucose infusion [29]. Frequent feeding was employed as the general treatment for all patients. Diazoxide served as the first line drug option, with unresponsiveness defined as inability to maintain glycemic control after 5 days at the maximum dose (range 5–15 mg/kg/day). In diazoxide-unresponsive patients, octreotide was administered, with unresponsiveness defined as inability to maintain glycemic control after 2 days at the maximum dose (range 5–25 μg/kg/day). For patients unresponsive to all conservative treatments, 18F-fluoro-L-dihydroxyphenylalanine computed tomography (^18^F-DOPA-PET/CT) scan was recommended, followed by surgical intervention based on focal or diffuse lesions identified through computed tomography (CT) scans.

### 2.2. Variants Analysis

This study received approval from the Ethics committee of Beijing children's hospital. A comprehensive panel for target enrichment was designed to include genes associated with CHI, namely, *ABCC8*, *KCNJ11*, *GLUD1*, *GCK*, *HADH*, uncoupling protein 2 (*UCP2*), *HNF1A*, *HNF4A*, phosphoglucomutase 1 (*PGM1*), phosphomannomutase 2 (*PMM2*), hexokinase 1 (*HK1*), potassium voltage-gated channel subfamily Q member 1 (*KCNQ1*), *SLC16A1*, forkhead box A2 (*FOXA2*), eukaryotic translation initiation factor 2 subunit gamma (*EIF2S3*), *and* calcium voltage-gated channel subunit alpha1 D (*CACNA1D*), sourced from the RefSeq database (genome research consortium human build 37 [GRCh37]/human genome build 19 [hg19]). All genes underwent sequenced by the next generation sequencing assay. Variants were annotated using the ANNOVAR software (http://annovar.openbioinformatics.org/en/latest/) and cross-referenced with multiple databases, such as gnomAD, 1000 Genome, ESP6500, dbSNP, ExAC, ClinVar, and the human gene mutation database (HGMD). Three criteria were employed to select potential pathogenic variants in downstream analysis: (1) variants reads exceeding five, with a variant ration no less than 30%; (2) variants were excluded if their frequency exceeded 5% in gnomAD, ExAC, 1000 Genomes, and ESP6500 databases; and (3) the synonymous variants were excluded if not found in the HGMD database. Pathogenicity of genetic variants was assessed using the American College of Medical Genetics criteria and predicted by silico methods and tools like REVEL, SIFT, PolyPhen2, Mutation taster, and GERP++. All benign and likely benign variants were excluded from further consideration.

### 2.3. Statistical Analysis

International Business Machines Corporation Statistical Product Service Solutions (IBM SPSS) Statistics (22.0 Version; Armonk, NY, USA) was employed for data analysis. Counting variables are expressed as median (range) values, while categorical variables are presented as the number (*n*) and percentage (%) of patients. The Mann–Whitney *U* test and Student's *t* test were utilized to compare counting variables, and Pearson's chi-squared test, Yates's correction for continuity along with Fisher's exact test were applied to compare categorical variables. A *p* value < 0.05 was deemed statistically significant.

## 3. Results

### 3.1. General Characteristics

In this study, 121 non-consanguineous patients with monogenic CHI were recruited from 23 provinces, municipalities, or autonomous regions in China.  Table 1 and Table S1 provide an overview of the characteristics of all patients. Of the total, 37.2% were macrosomia at birth, and 50.4% occurring within the neonatal period. Severe symptoms (seizures and unconscious) were primary initial symptom in 62.8% of patients.

**Figure 1:**
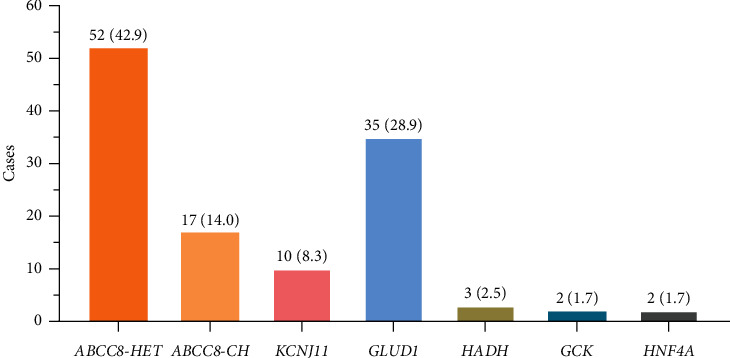
The spectrum of variants in 121 patients with monogenic CHI. The distribution of these patients is represented as number (*n*) and percentage (%). CH, compound heterozygote; CHI, congenital hyperinsulinism; HET, heterozygote.

As depicted in Figure 1, six CHI-related genes were identified (*ABCC8*, *KCNJ11*, *GLUD1*, *GCK*, *HADH*, and *HNF4A*). Heterozygous variants in *KATP* (*n* = 62) and *GLUD1* (*n* = 35) collectively account for 80.2%.

### 3.2. KATP Variants

#### 3.2.1. *ABCC8* Variants

*ABCC8* variants were identified in 69 (57.0%) patients. Among these, 52 were heterozygous. Analysis of both parents for 51 probands revealed 42 paternally inherited, 3 maternally inherited, and 6 de novo variants. The remaining 17 patients were compound heterozygotes. A total of 70 different variants were observed, including 20 novel variants, comprising 36 missense, 14 nonsense, 12 frame-shift, and 8 splicing variants.

Of the 52 heterozygous patients, 25.0% (13/52) were diazoxide-responsive, while 38.5% (20/52) exhibited responsiveness to octreotide. Only one achieved glycemic control with frequent feeding, carrying the maternally inherited variant (p.G1478R) which has been already reported [30]. The *ABCC8* variants (p.L580_S581insFASL and p.S986*⁣*^*∗*^) found in two patients, respectively, were previously reported to be associated with diabetes. Ten paternally inherited patients who were unresponsive to non-surgical treatment accepted surgery referring to eight focal CHI and two diffuse CHI (the flowchart of therapeutic options shown in Figure S1). Nine patients abandoned any form of therapy, resulting in five lost to follow-up, three mortalities, and one with irreversible brain damage eventually.

Among compound heterozygotes, three treated with diazoxide, eight with octreotide, and two underwent pancreatectomy owing to diffuse CHI. Four patients forwent any treatment, resulting in two mortalities and two cases of brain damage.

The high-frequency variants (p.G111R) were identified in seven heterozygotes and three compound heterozygotes. All of them were diazoxide-unresponsive, but half exhibited responsiveness to octreotide, two gave up any treatment (one mortality and another with irreversible brain damage). Surgical intervention was pursued in two heterozygous patients with focal CHI and one compound heterozygous patient with diffuse CHI.

#### 3.2.2. *KCNJ11* Variants

Ten patients (8.3%) carried heterozygous *KCNJ11* variants with eight paternally inherited, one maternally inherited, and one of unknown origin. A total of nine different variants were identified, including three novel variants, consisting of seven missense variants and two nonsense variants.

Diazoxide showed effectiveness in two patients, while octreotide proved efficacious in five. Pancreatectomy was performed in two cases due to diffuse CHI. Only one patient gave up, experiencing spontaneous remission at 7 years old in spite of developmental delay.

### 3.3. Non-*KATP* Variants

#### 3.3.1. *GLUD1* Variants

A total of 35 patients (35/121, 28.9%) presented with heterozygous *GLUD1* variants, with the majority (32/35, 91.4%) harboring *de novo* variants. The remaining three patients all denied familial history of hyperinsulinism and/or diabetes, with two paternally inherited and one maternally inherited. Eleven previously reported missense mutations were detected, distributed across exons 6, 7, 11, and 12. The high-frequency variants (p.S498L), found in 12/35 (34.3%) patients, resides in exon 11. Thirty-one patients (31/35, 88.6%) presented with hyperammonemia, as four patients exhibited normal concentration of serum ammonia in the initial evaluation. Only four patients occurred during neonatal period, with variants residing in exon 11 or 12. Twenty-nine patients depended on diazoxide to keep glycemic control, the remaining six carried variants located in exon 6 or 7 and merely required frequent feeding. These observations prompted us to conducted subgroup analysis based on the distribution of exons.

#### 3.3.2. Other Variants

As detailed in Table 1 and Table S1, three patients (2.5%) had variants in *HADH* gene, 2 (1.7%) in *GCK*. Additionally, two previously reported *HNF4A* variants, p.R63W and p.V382I, associated with maturity-onset diabetes of the young (MODY) [31, 32], were found in two patients (1.7%) without family history of hyperinsulinism and/or diabetes. Among these seven diazoxide-responsive patients, four had neonatal onset time and five presented seizures initially.

### 3.4. Genotype–Phenotype Correlations

Neither the comparison of clinical features between patients with variants in *ABCC8* and *KCNJ11* genes nor that between patients with *ABCC8* variants (p.G111R) and other *ABCC8* variants had significant difference (seen in Tables S2 and S3). As shown in Table 1, patients with *KATP* variants tended to be macrosomia at birth, had earlier onset time, exhibited a lower response rate to non-surgical interventions, while displayed a higher level of serum C-peptide than those with *GLUD1* variants. Additionally, patients with *GLUD1* variants had severer symptoms at onset than those with *KATP* variants.

The subgroup analyses were presented in Tables 2 and 3. For *KATP* subgroup, neonatal-onset patients tended to be macrosomia, had a higher birth weight, inclined towards milder symptoms at onset, exhibited a higher proportion of surgical intervention, and had higher concentrations of serum insulin and C-peptide than those with non-neonatal onset. For *GLUD1* subgroup, patients with variants in exons 6 and 7 demonstrated later onset compared to those in exons 11 and 12, with a higher response rate of frequent feeding.

## 4. Discussion

We conducted an in-depth analysis of clinical features in a monogenic CHI cohort containing 121 patients cross mainland China. Currently, our cohort is the largest sample size with monogenic CHI from a single center in China. Our findings highlight the genetic patterns as well as attention on neonatal-onset cases, underscoring the irreplaceability of targeted next-generation sequencing (tNGS) for management of CHI and providing evidence for the efficacy of non-surgical therapy in this disease.

The two significant discrepancies observed in our cohort are deficiency of homozygous *KATP* variants and a relatively high prevalence of *GLUD1* variants. In various studies (summarized in Table S4), variants in *ABCC8* have consistently accounted for the predominant genetic cause, explaining 55%–100% of positive variant cases, followed by *KCNJ11* or *GLUD1* [7–9, 33, 34]. Notably, the subjects in most of these studies are not Chinese, but rather European or American populations with high rates of consanguinity and ethnic migration. The percentage of *GLUD1* variants confirmed in our cohort was much higher than the Caucasian populations and analogous to findings from prior studies in South China and Japan [15, 34]. The ethnic variabilities shown in molecular spectrum of monogenic CHI indicates a potentially characteristic among the Chinese population.

In the present investigation, we noted that response rate of non-surgical therapy reached 65.9% (52/79) in patients with *KATP* variants, and 100% (42/42) in those with non-*KATP* variants, totaling 77.7%. Long-term follow-up of conservatively treated patients revealed spontaneous remission occurring in 48%–68% of cases over time [21, 35]. Ludwig et al. [36] found that approximately 15% of CHI patients may experience permanent cognitive defects possibly caused by delayed diagnosis. Moreover, the prognosis of operation is not as favorable as anticipated. Diffuse CHI accounts for 60% to 70% of cases [37]. Given the need to remove 90% to 98% of the pancreas, challenges arise in maintaining glycemic control after surgery, followed by certain rates of recurrence and reoperation [19, 38]. The outcomes show that 25%–90% of patients will inevitably progress to insulin-dependent diabetes, with approximately 15% also facing exocrine pancreatic dysfunction [19, 21, 38, 39]. Theoretically, focal CHI can be alleviated through partial resection with an actual cure rate of 95%. Approximately 70% of the focal lesions reside in the head or the uncus of the pancreas, and Roux-en-Y pancreatico-jejunostomy has been recommended for such cases. The postoperative complications, particularly pancreatic fistulas, can cause technical difficulties in open or laparoscopic approach for pediatric surgeons. Therefore, surgical intervention requires careful consideration and a non-surgical approach is increasingly preferred in several centers [40–42]. Our previous work has detailed the efficacy of octreotide for diazoxide-unresponsive CHI patients with *KATP* variants [43]. Coincidentally, Yorifuji et al. [29] demonstrated a higher response rate in such diazoxide-unresponsive cases among the Japanese population. Coupled with the unique genetic patterns in our observations, we lean towards favoring non-surgical therapy during recent years, collectively.

Only one feeding-responsive patient carrying the variant (p.G1478R) from his mother that Casertano et al. [30] described in two diazoxide-unresponsive siblings but progressed to diabetes without operative intervention at 15-years old and 20-years old, respectively. Recent studies have shown that carriers of *ABCC8* variants may develop diabetes during adulthood, even including homozygotes [44, 45]. Besides, two patients carried heterozygous *ABCC8* variants from their respectively unaffected fathers and accepted octreotide treatment, one having the variant (p.L580_S581insFASL) that Wang et al. [46] documented as a *de novo* variant in a cohort of diabetes, another one carrying the variant (p.S986*⁣*^*∗*^) that Moalla et al. [47] reported in a 19-year male with diabetes. It is unknown whether these patients with diabetes have experienced hypoglycemia in childhood. This observation suggests potential progression from CHI to diabetes owing to the possible mechanism of apoptosis of the *β*-cells [48]. Present visit did not find these patients in our cohort have shown propensity for hyperglycemia. Although some carriers of *KATP* variants do not respond to diazoxide, non-surgical treatment should be pursued to avoid surgery as much as possible due to the high risk of diabetes over time.

A high frequency of *ABCC8* variants (p.G111R) were identified in 10 diazoxide-unresponsive patients, suggestive of a potential founder effect. The variants (p.G111R) have been previously documented in the Asian Indian (summarized in Table S5) and other reports being isolated cases [49]. Hence, we reasonably postulated that the founder variants in *ABCC8* display geographical diversities among different populations and the variant (p.G111R) may represents a potential founder effect in children from an Asian background [9, 15, 50]. Through our summary, only one heterozygote received diazoxide treatment, a majority of affected children displayed responsiveness to somatostatin analogs, even including compound heterozygotes and homozygotes. For patients with the variant (p.G111R), especially Chinese children, non-surgical treatment rather than operation should be recognized initially.

The clinical characteristics of *GLUD1* variants in 35 patients were consistent with those reported in previous literatures, as well as the high-frequency variant (p.S498L) [25, 51, 52]. It is noteworthy that patients with *GLUD1* variants in exons 11 and 12 were associated with earlier onset and diazoxide treatment. Among the six patients with frequent feeding, carrying variants located in exons 6 and 7, which mirrors recent evidence [51, 53, 54]. Then we speculated that *GLUD1* variants from different exons might indicate the onset time and disease severity. This may be attributed to the encoding of a unique allosteric domain in exons 11 and 12, wherein active variants result in increased activity of glucose dehydrogenase and severe symptoms [55].

We found that neonatal-onset patients with *KATP* variants were prone to be macrosomia at birth, presented with milder symptoms and had a higher percentage of operative intervention, along with higher levels of serum insulin and C-peptide than non-neonatal onset ones. It is obvious that severity of symptoms may not be related to secretions of serum insulin and C-peptide. Our intention is to highlight that therapeutic choice, particular surgery, depends on the genotypes and onset time possibly due to severer dysfunction of the pancreatic *β*-cells. This result may help improving patient–clinician communication about the benefits and risks of treatment to refine shared decision making for children with CHI.

## 5. Limitations and Outlook

On the one hand, our analysis merely focused on the early stage of CHI, long-term follow-up and outcomes of these patients are pending to supplement in the future. Moreover, panel for the older patients did not include some of these genes (e.g., *HK1*, *KCNQ1*, *FOXA2*, *and EIF2S3*), which means that we have underestimated the number of positive cases. On the other hand, given that sirolimus is predominantly documented in case reports or case series and there is a lack of well-designed cohort studies. The majority of patients in a 5-year follow-up stopped sirolimus due to complications [56–59]. We endeavored to persuade some parents to consider sirolimus; however, they declined due to concerns about potential side effects and uncertainties surrounding long-term outcomes. Moreover, a systematic review of nifedipine have suggested its ineffectiveness in patients with *KATP* variants [60]. Consequently, our center exercises considerable caution regarding the use of medications outside of diazoxide and somatostatin analogs, resulting in a limited exploration of these alternative drugs.

## 6. Conclusion

*ABCC8* variants have always elucidated the primary genetic cause of patients with monogenic CHI. Two factors in our substantial cohort, the deficiency of homozygous variants in *KATP* genes and a rather high prevalence of *GLUD1* variants within the Chinese population stand out prominently, contributing for non-surgical therapy. The variants associated with diabetes from the literature, while causing CHI in our cohort, may expand the phenotypic spectrum, and serve as additional considerations for us to avoid surgical intervention as much as possible. Our results suggest that neonatal-onset children should be paid attention owing to relatively low response rate of non-surgical treatment, especially those with *KATP* variants.

## Figures and Tables

**Table 1:** Clinical characteristics of 121 patients with monogenic CHI.

Clinical variables	All patients	*KATP* channel	*GLUD1*	Other variants	*p* value^a^
Cases, *n* (%)	121	79 (65.3)	35 (28.9)	7 (5.8)	—
Gender, M/F (%)	57.9/42.1	53.2/46.8	62.9/37.1	85.8/14.2	0.336
Macrosomia, *n* (%)	45 (37.2)	39 (49.4)	4 (11.4)	2 (28.6)	<0.001
Birth weight (kg)	3.65 (1.80–5.50)	3.89 (1.80–5.50)	3.50 (2.64–4.50)	3.80 (3.20–4.81)	<0.001
Severe symptoms at onset, *n* (%)	76 (62.8)	38 (48.1)	33 (94.2)	5 (71.4)	<0.001
Days at presentation, days	26 (1–2792)	2 (1–690)	210 (1–2792)	26 (1–360)	<0.001
Neonatal-onset, *n* (%)	61 (50.4)	53 (67.1)	4 (11.4)	4 (57.1)	—
Non-neonatal onset, *n* (%)	60 (49.6)	26 (32.9)	31 (88.6)	3 (42.9)	—
Serum insulin (mU/L)	11.20 (1.66–110.60)	13.29 (1.66–110.6)	9.56 (3.10–89.55)	10.24 (4.29–37.70)	0.443
Serum C-peptide (nmol/L)	2.84 (0.6–13.98)	3.12 (0.60–9.17)	2.20 (0.69–13.98)	2.72 (0.92–6.13)	0.018
Hyperammonemia, *n* (%)	40 (33.1)	9 (11.4)	31 (88.6)	n.p.	<0.001
Therapeutic options, *n* (%)	—	—	—	—	0.011^b^
Frequent feeding	7 (5.8)	1 (1.3)	6 (17.1)	n.p.	—
Diazoxide treatment	54 (44.6)	18 (22.8)	29 (82.9)	7 (100.0)	—
Octreotide treatment	33 (27.3)	33 (41.8)	n.p.	n.p.	—
Surgery (focal)	8 (6.6)	8 (10.1)	n.p.	n.p.	—
Surgery (diffuse)	6 (5.0)	6 (7.6)	n.p.	n.p.	—
Abandon treatment	13 (10.7)	13 (16.4)	n.p.	n.p.	—
Diazoxide dose (mg/kg/day)	5.5 (3.5–12.5)	6.2 (3.8–11.3)	5.0 (3.5–12.5)	7.3 (4.0–10.4)	0.254

Abbreviations: CHI, congenital hyperinsulinism; F, female; M, male; n.p., no patient.

^a^Only *KATP* channel subgroup and *GLUD1* subgroup were compared, cause of the limited number of patients with variants in rare genes.

^b^We made comparison based on responsiveness of conservative treatment, excluding patients who have discontinued treatment. The statistical methods used in other tables are consistent with those used in this table. Comparisons of counting variables were performed by the Mann–Whitney *U* test, and categorical variables were performed by the Pearson's chi-squared test or Yates's correction for continuity.

**Table 2:** Clinical characteristics of 79 patients with *KATP* channel variants.

Clinical variables	Neonatal-onset	Non-neonatal onset	*p* value
Cases, *n* (%)	53 (67.1)	26 (32.9)	—
Gender, M/F (%)	52.8/47.2	53.8/46.2	0.932
Macrosomia, *n* (%)	33 (62.3)	6 (23.1)	<0.001
Birth weight (kg)	4.20 (2.52–5.50)	3.38 (1.80–4.90)	<0.001
Severe symptoms at onset, *n* (%)	17 (32.1)	21 (80.7)	<0.001
Serum insulin (mU/L)	18.35 (1.66–110.60)	7.96 (2.39–36.60)	<0.001
Serum C-peptide (nmol/L)	3.92 (1.19–9.17)	2.35 (0.60–4.80)	<0.001
Hyperammonemia, *n* (%)	5 (9.4)	4 (15.4)	0.685
Therapeutic options, *n* (%)	—	—	0.007
Frequent feeding	n.p.	1 (3.8)	—
Diazoxide treatment	13 (24.5)	5 (19.2)	—
Octreotide treatment	19 (35.8)	14 (53.8)	—
Surgery (focal)	7 (13.2)	1 (3.8)	—
Surgery (diffuse)	6 (11.3)	n.p.	—
Abandon treatment	8 (15.2)	5 (19.4)	—
Diazoxide dose (mg/kg/day)	6.7 (4.7–11.3)	5.1 (3.8–7.0)	0.176
Octreotide dose (ug/kg/day)	12.5 (5.0–25.0)	10.0 (3.8–22.5)	0.103

*Note:* Counting variables represented as median (range) values. Categorical variables are represented as the number (*n*) and percentage (%) of the patients. Comparisons of counting variables were performed by the Mann–Whitney *U* test or Student's *t* test, and comparisons of categorical variables were performed by Pearson's chi-squared test or Yates's correction for continuity.

Abbreviations: F, female; M, male; n.p., no patient.

**Table 3:** Clinical characteristics of patients with *GLUD1* variants based on different exons.

Clinical variables	Variants of exon 6/7	Variants of exon 11/12	*p* value
Cases, *n* (%)	18 (51.4)	17 (48.6)	—
Gender, M/F (%)	55.6/44.4	70.6/29.4	0.489
Macrosomia, *n* (%)	2 (11.1)	2 (11.8)	0.677
Birth weight (kg)	3.55 (2.70–4.50)	3.45 (2.64–4.10)	0.146
Severe symptoms at onset, *n* (%)	18 (100.0)	15 (94.2)	0.229
Days at presentation, days	360 (112–436)	126 (1–420)	—
Serum insulin (mU/L)	10.5 (3.5–73.9)	10.5 (3.9–89.6)	0.325
Serum C-peptide (nmol/L)	2.0 (1.0–6.1)	2.5 (1.5–14.0)	0.069
Normal ammonia, *n* (%)	3 (16.7)	1 (5.9)	0.603
Therapeutic options, *n* (%)	—	—	0.019
Frequent feeding	6 (33.3)	n.p.	—
Diazoxide treatment	12 (66.7)	17 (100.0)	—
Diazoxide dose (mg/kg/day)	4.6 (3.5–10.0)	5.4 (3.8–8.9)	0.175

*Note:* Comparisons of counting variables were performed by the Mann–Whitney *U* test, and comparisons of categorical variables were performed by Fisher's exact test.

Abbreviations: F, female; M, male; n.p., no patient.

## Data Availability

The data in this study are available from the corresponding author upon reasonable request.

## Nomenclature

CHI: Congenital hyperinsulinism
KATP: ATP-sensitive potassium channel
^18^F-DOPA-PET/CT: ^18^F-fluoro-L-dihydroxyphenylalanine computed tomography
tNGS: Targeted next-generation sequencing
LGA: Large for gestational age
AGA: Appropriate for gestational age
MODY: Maturity-onset diabetes of the young.

## Supporting Information

Supporting Information Table S1: summary of 121 patients with monogenic congenital hyperinsulinism. Table S2: clinical characteristics of 79 patients with *ABCC8* and *KCNJ11* variants. Table S3: clinical characteristics of 69 patients with *ABCC8* variants. Table S4: spectrum of positive variant patients with monogenic CHI from different cohort. Table S5: summary of patients with *ABCC8* variant (p.G111R) reported previously. Figure S1: the flowchart of therapeutic options of 121 patients based on genotypes. The number of patients of each genotype is represented as number (*n*). CH, compound heterozygote; HET, heterozygote.
