# Supplementary material for: Non-surgical Treatment May be Appropriate for Most Chinese Children With Monogenic Congenital Hyperinsulinism Based on a Retrospective Study of 121 Patients
Source: Pediatr Diabetes. 2024 Nov 19;2024:3961900. doi: 10.1155/2024/3961900 (PMC12016755; doi:10.1155/2024/3961900)
Supplement: Supporting Information — Table S1: summary of 121 patients with monogenic congenital hyperinsulinism. Table S2: clinical characteristics of 79 patients with ABCC8 and KCNJ11 variants. Table S3: clinical characteristics of 69 patients with ABCC8 variants. Table S4: spectrum of positive variant patients with monogenic CHI from different cohort. Table S5: summary of patients with ABCC8 variant (p.G111R) reported previously. Figure S1: the flowchart of therapeutic options of 121 patients based on genotypes. The number of patients of each genotype is represented as number (n). CH, compound heterozygote; HET, heterozygote. [file 3961900.f1.docx]

**Supplementary Table S1.** Summary of 121 patients with monogenic congenital hyperinsulinism.

| Case | Gene | cDNA | Amino acid | status | Origin | Brith  delivery | Brith  weight (kg) | Onset  (days) | Symptom | Therapeutic  option |
| --- | --- | --- | --- | --- | --- | --- | --- | --- | --- | --- |
| ***ABCC8*** (NM_000352.6) | | | | | | | | | | |
| 1 | exon 1 | c.18C>A | p.C6* | het | paternal | term | 4.2 | 150 | seizure | OCT |
| 2 | exon 1 | c.86A>G | p.D29G | het | paternal | term | 3.4 | 80 | seizure | refuse |
| 3 | intron 1 | c.149-2A>C | p.? | het | paternal | term | 3.7 | 3 | seizure | OCT |
| 4 | exon 2 | c.216C>A | p.N72K | het | paternal | term | 4.56 | 1 | none | DZX |
| 5 | exon 2 | c.276-277insCATC | p.I93Hfs*3 | het | paternal | term | 3.6 | 3 | seizure | DZX |
| 6 | exon 3 | c.331G>A | p.G111R | het | paternal | term | 4.8 | 1 | none | OCT |
| 7 | exon 3 | c.331G>A | p.G111R | het | paternal | term | 3.3 | 2 | seizure | focal |
| 8 | exon 3 | c.331G>A | p.G111R | het | paternal | preterm | 2.52 | 2 | lethargy | refuse |
| 9 | exon 3 | c.331G>A | p.G111R | het | paternal | preterm | 4.4 | 1 | none | refuse |
| 10 | exon 3 | c.331G>A | p.G111R | het | paternal | term | 3 | 124 | unconscious | OCT |
| 11 | exon 3 | c.331G>A | p.G111R | het | paternal | term | 3.5 | 2 | seizure | focal |
| 12 | exon 3 | c.331G>A | p.G111R | het | paternal | term | 3.6 | 162 | seizure | OCT |
| 13 | exon 3 | c.428G>A | p.W143* | het | paternal | term | 3 | 240 | seizure | OCT |
| 14 | exon 4 | c.428G>A | p.W143* | het | paternal | term | 4.2 | 2 | seizure | refuse |
| 15 | exon 4 | c.428G>A | p.W143* | het | paternal | term | 3.15 | 3 | lethargy | focal |
| 16 | exon 4 | c.563A >G | p.N188S | het | paternal | term | 4.76 | 3 | lethargy | refuse |
| 17 | exon 5 | c.683G>A | p.E228D | het | paternal | term | 4.05 | 1 | seizure | focal |
| 18 | exon 5 | c.752G>A | p.G251E | het | paternal | term | 4.75 | 2 | seizure | OCT |
| 19 | exon 6 | c.850dupG | p.A284fs | het | paternal | term | 3.8 | 2 | seizure | focal |
| 20 | exon 7 | c.1108A>T | p.R370W | het | paternal | term | 4.1 | 90 | seizure | OCT |
| 21 | intron 7 | c.1176+1G>A | p.? | het | paternal | term | 3.8 | 1 | none | focal |
| 22 | exon 10 | c.1473T>G | p.Y491* | het | de novo | term | 3.2 | 60 | seizure | DZX |
| 23 | exon 10 | c.1501G>A | p.E501K | het | maternal | term | 4.6 | 3 | lethargy | DZX |
| 24 | exon 10 | c.1508T>C | p.L503P | het | paternal | term | 4.1 | 3 | seizure | DZX |
| 25 | exon 10 | c.1585G>A | p.E529K | het | paternal | term | 3.33 | 2 | lethargy | OCT |
| 26 | exon 12 | c.1730_1741dupTTGCCT  CCCTCT | p.L580_S581insFASL | het | paternal | term | 4.4 | 2 | seizure | OCT |
| 27 | exon 12 | c.1792C>T | p.R598* | het | paternal | term | 4.4 | 1 | seizure | refuse |
| 28 | exon 12 | c.1792C>T | p.R598* | het | paternal | term | 3.3 | 240 | lethargy | DZX |
| 29 | intron 14 | c.2041-21G>A | p.? | het | paternal | term | 5.04 | 2 | lethargy | diffuse |
| 30 | exon 16 | c.2144T＞G | p.V715K | het | paternal | term | 4 | 1 | none | OCT |
| 31 | exon 21 | c.2521C>G | p.R841G | het | paternal | term | 4 | 175 | seizure | OCT |
| 32 | exon 21 | c.2521C>T | p.R841* | het | paternal | term | 3.3 | 236 | seizure | refuse |
| 33 | exon 23 | c.2800C>T | p.Q934* | het | paternal | term | 2.85 | 120 | seizure | OCT |
| 34 | exon 25 | c.2975C>A | p.S986* | het | paternal | preterm | 3.59 | 1 | lethargy | OCT |
| 35 | exon 29 | c.3640C>T | p.R1214W | het | de novo | term | 3.65 | 312 | lethargy | DZX |
| 36 | exon 30 | c.3663_3664insG | p.F1222Vfs*184 | het | paternal | term | 4.9 | 90 | seizure | OCT |
| 37 | exon 32 | c.3888G>A | p.W1296* | het | paternal | term | 3.7 | 1 | lethargy | DZX |
| 38 | exon 33 | c.4078G>T | p.V1360L | het | de novo | preterm | 4.19 | 1 | none | OCT |
| 39 | exon 34 | c.4132G>A | p.G1378S | het | maternal | term | 3.89 | 2 | lethargy | OCT |
| 40 | exon 34 | c.4176C>G | p.F1392L | het | de novo | term | 4.2 | 1 | lethargy | DZX |
| 41 | exon 35 | c.4252C>T | p.R1418C | het | paternal | term | 5.08 | 1 | none | diffuse |
| 42 | exon 36 | c.4370G>T | p.A1457V | het | de novo | term | 4.9 | 1 | none | DZX |
| 43 | exon 36 | c.4374G>C | p.Q1458H | het | n.d. | term | 3.15 | 163 | lethargy | OCT |
| 44 | exon 37 | c.4432G>A | p.G1478R | het | maternal | term | 4.9 | 690 | lethargy | feeding |
| 45 | exon 37 | c.4463A>G | p.Q1488R | het | de novo | term | 4.7 | 1 | none | DZX |
| 46 | intron 11 | IVS11+2T＞C | p.? | het | paternal | term | 3.64 | 2 | seizure | OCT |
| 47 | exon 37 | c.4477G>A | p.A1493W | het | paternal | term | 3.6 | 139 | seizure | OCT |
| 48 | exon 37 | c.4477G>A | p.A1493W | het | paternal | term | 3.48 | 3 | lethargy | focal |
| 49 | exon 37 | c.4478G>A | p.R1493Q | het | paternal | term | 5.12 | 1 | none | refuse |
| 50 | exon 37 | c.4478G>A | p.R1493Q | het | paternal | term | 4.6 | 1 | seizure | DZX |
| 51 | exon 39 | c.4612C>T | p.R1538* | het | paternal | term | 4.9 | 30 | seizure | focal |
| 52 | exon 39 | c.4661G>A | p.G1554D | het | paternal | preterm | 3.6 | 15 | lethargy | DZX |
| 53 | exon 1 | c.106C>T | p.H36Y | ch | paternal | term | 4.55 | 1 | none | OCT |
|  | exon 35 | c.4304T>A | p.I1435N | ch | maternal |  |  |  |  |  |
| 54 | exon 3 | c.331G>A | p.G111R | ch | maternal | term | 4.22 | 1 | none | OCT |
|  | exon 39 | c.4612C>T | p.R1538* | ch | paternal |  |  |  |  |  |
| 55 | exon 3 | c.331G>A | p.G111R | ch | paternal | term | 4.9 | 1 | none | OCT |
|  | exon 12 | c.1792C>T | p.R598* | ch | maternal |  |  |  |  |  |
| 56 | exon 3 | c.382G>A | p.E128K | ch | maternal | preterm | 3.26 | 1 | none | diffuse |
|  | exon 15 | c.2078delinsCC | p.I1693Tfs*52 | ch | paternal |  |  |  |  |  |
| 57 | exon 5 | c.752G>A | p.G251E | ch | paternal | term | 3.15 | 202 | seizure | DZX |
|  | exon 10 | c.1585-1587delGAC | p.E529del | ch | maternal |  |  |  |  |  |
| 58 | exon 6 | c.863G>A | p.W288* | ch | paternal | term | 3.65 | 120 | unconscious | refuse |
|  | exon 21 | c.2506C>T | p.R836* | ch | maternal |  |  |  |  |  |
| 59 | exon 9 | c.1413_1414insCGCT | p.P472Gfs*24 | ch | paternal | term | 3.8 | 1 | none | OCT |
|  | exon 7 | c.1174C>T | p.Q392* | ch | maternal |  |  |  |  |  |
| 60 | exon 13 | c.1894del | p.Q632Rfs*15 | ch | maternal | term | 4.2 | 1 | seizure | DZX |
|  | exon 28 | c.3557G>A | p.R1186K | ch | paternal |  |  |  |  |  |
| 61 | intron 13 | c.1923+1G>A | p.? | ch | maternal | preterm | 3.78 | 1 | lethargy | refuse |
|  | intron 32 | c.3989-9G>A | p.? | ch | paternal |  |  |  |  |  |
| 62 | exon 21 | c.2506C>T | p.R836* | ch | paternal | term | 5.5 | 1 | none | refuse |
|  | exon 28 | c.3540C>G | p.Y1180* | ch | maternal |  |  |  |  |  |
| 63 | intron 24 | c.2921-6C>T | p.? | ch | paternal | preterm | 4.7 | 1 | seizure | OCT |
|  | exon 36 | c.4353T>C | p.L1451P | ch | maternal |  |  |  |  |  |
| 64 | exon 25 | c.3124_c.3126delACCins  CAGCCAGGAACTG | p.T1042Qfs*75 | ch | paternal | term | 4.4 | 10 | lethargy | OCT |
|  | exon 24 | c.2832_c.2833insA | p.E945Rfs*25 | ch | maternal |  |  |  |  |  |
| 65 | exon 29 | c.3632T>C | p.L1211P | ch | paternal | preterm | 1.8 | 10 | lethargy | refuse |
|  | exon 9 | c.1412C>T | p.A471V | ch | maternal |  |  |  |  |  |
| 66 | exon 29 | c.3650G >A | p.R1217K | ch | paternal | term | 3.15 | 460 | seizure | DZX |
|  | exon 14 | c.1990C >T | p.Q664* | ch | maternal |  |  |  |  |  |
| 67 | exon 30 | c.3736T>C | p.W1246R | ch | paternal | preterm | 3.56 | 1 | lethargy | OCT |
|  | exon 4 | c.536A>G | p.Y179C | ch | maternal |  |  |  |  |  |
| 68 | exon 7 | c.1032del | p.F345Lfs*14 | ch | paternal | term | 4.75 | 2 | seizure | OCT |
|  | exon 15 | c.2113del | p.R705Efs*4 | ch | maternal |  |  |  |  |  |
| 69 | exon 3 | c.331G>A | p.G111R | ch | paternal | term | 4.63 | 1 | none | diffuse |
|  | intron 12 | c.1817+2T＞C | p.? | ch | maternal |  |  |  |  |  |
| ***KCNJ11*** (NM_000525.4) | | | | | | | | | | |
| 70 | exon 1 | c.101G>A | p.R34H | het | paternal | term | 3 | 92 | seizure | OCT |
| 71 | exon 1 | c.400G>C | p.G134R | het | paternal | preterm | 4.28 | 1 | none | diffuse |
| 72 | exon 1 | c.407G>T | p.R136L | het | n.d. | preterm | 3.2 | 1 | none | DZX |
| 73 | exon 1 | c.413T>A | p.V138E | het | paternal | term | 3.5 | 143 | seizure | OCT |
| 74 | exon 1 | c.413T>A | p.V138E | het | paternal | term | 4 | 2 | seizure | diffuse |
| 75 | exon 1 | c.488T>C | p.M163T | het | paternal | term | 4 | 1 | none | DZX |
| 76 | exon 1 | c.703C>T | p.Q235* | het | maternal | term | 4.55 | 1 | none | OCT |
| 77 | exon 1 | c.844G>A | p.E282K | het | paternal | term | 3.36 | 92 | seizure | OCT |
| 78 | exon 1 | c.881C>T | p.T294M | het | paternal | term | 3.2 | 37 | unconscious | refuse |
| 79 | exon 1 | c.895C>T | p.Q299* | het | paternal | term | 3.4 | 140 | seizure | OCT |
| ***GLUD1*** (NM_005271) | | | | | | | | | | |
| 80 | exon 6 | c.820C>T | p.R274C | het | maternal | term | 3.1 | 112 | seizure | DZX |
| 81 | exon 6 | c.820C>T | p.R274C | het | de novo | term | 3.3 | 383 | seizure | DZX |
| 82 | exon 6 | c.820C>T | p.R274C | het | de novo | term | 3.7 | 382 | seizure | DZX |
| 83 | exon 6 | c.820C>T | p.R274C | het | de novo | term | 4.5 | 450 | seizure | feeding |
| 84 | exon 6 | c.820C>T | p.R274C | het | de novo | term | 3.8 | 318 | seizure | DZX |
| 85 | exon 6 | c.820C>T | p.R274C | het | de novo | term | 3.65 | 120 | seizure | feeding |
| 86 | exon 6 | c.820C>T | p.R274C | het | de novo | term | 3.3 | 210 | seizure | DZX |
| 87 | exon 6 | c.820C>T | p.R274C | het | de novo | term | 3 | 243 | seizure | DZX |
| 88 | exon 7 | c.943C>T | p.H315Y | het | de novo | term | 3.5 | 360 | seizure | DZX |
| 89 | exon 7 | c.943C>T | p.H315Y | het | de novo | term | 3.5 | 360 | seizure | DZX |
| 90 | exon 7 | c.953G>A | p.R318K | het | de novo | term | 3.5 | 180 | seizure | DZX |
| 91 | exon 7 | c.964C>T | p.R322C | het | de novo | term | 2.7 | 780 | seizure | feeding |
| 92 | exon 7 | c.965G>A | p.R322H | het | de novo | term | 3.6 | 436 | seizure | DZX |
| 93 | exon 7 | c.965G>A | p.R322H | het | paternal | term | 3.8 | 1080 | seizure | feeding |
| 94 | exon 7 | c.965G>A | p.R322H | het | de novo | term | 3.6 | 100 | seizure | feeding |
| 95 | exon 7 | c.965G>A | p.R322H | het | de novo | term | 3.35 | 2792 | seizure | feeding |
| 96 | exon 7 | c.965G>A | p.R322H | het | de novo | term | 4.25 | 420 | seizure | DZX |
| 97 | exon 7 | c.965G>A | p.R322H | het | de novo | term | 3.9 | 360 | seizure | DZX |
| 98 | exon 11 | c.1493C>T | p.S498L | het | de novo | term | 3.2 | 151 | seizure | DZX |
| 99 | exon 11 | c.1493C>T | p.S498L | het | de novo | term | 3 | 82 | seizure | DZX |
| 100 | exon 11 | c.1493C>T | p.S498L | het | de novo | term | 2.9 | 420 | seizure | DZX |
| 101 | exon 11 | c.1493C>T | p.S498L | het | de novo | term | 3.7 | 171 | seizure | DZX |
| 102 | exon 11 | c.1493C>T | p.S498L | het | de novo | term | 2.64 | 204 | seizure | DZX |
| 103 | exon 11 | c.1493C>T | p.S498L | het | de novo | preterm | 3.24 | 126 | seizure | DZX |
| 104 | exon 11 | c.1493C>T | p.S498L | het | paternal | term | 3.3 | 6 | seizure | DZX |
| 105 | exon 11 | c.1493C>T | p.S498L | het | de novo | term | 3.5 | 28 | seizure | DZX |
| 106 | exon 11 | c.1493C>T | p.S498L | het | de novo | term | 4 | 150 | seizure | DZX |
| 107 | exon 11 | c.1493C>T | p.S498L | het | de novo | term | 3.5 | 240 | seizure | DZX |
| 108 | exon 11 | c.1493C>T | p.S498L | het | de novo | term | 3.65 | 240 | seizure | DZX |
| 109 | exon 11 | c.1493C>T | p.S498L | het | de novo | term | 3.5 | 90 | seizure | DZX |
| 110 | exon 11 | c.1495C>A | p.G499C | het | de novo | term | 2.8 | 1 | lethargy | DZX |
| 111 | exon 12 | c.1496G>A | p.G499D | het | de novo | term | 3.6 | 1 | none | DZX |
| 112 | exon 12 | c.1501T>C | p.S501P | het | de novo | term | 3.45 | 180 | seizure | DZX |
| 113 | exon 12 | c.1516G>A | p.V506M | het | de novo | term | 4.1 | 71 | seizure | DZX |
| 114 | exon 12 | c.1519G>A | p.H507Y | het | de novo | term | 2.8 | 240 | seizure | DZX |
| ***HADH*** (NM_005327.7 ) | | | | | | | | | | |
| 115 | exon 4 | c.438C>A | p.S146R | ch | paternal | term | 3.2 | 26 | seizure | DZX |
|  | exon 7 | c.773C>T | p.P258L |  | maternal |  |  |  |  |  |
| 116 | exon 5 | c.613delG | p.G205Efs*13 | ch | maternal | term | 3.2 | 173 | seizure | DZX |
|  | intron 3 | c.420-1G>T | p.? |  | paternal |  |  |  |  |  |
| 117 | exon 1 | c.89T>A | p.V30E | ch | maternal | term | 3.8 | 360 | seizure | DZX |
|  | exon 3 | c.317C>T | p.A106V |  | paternal |  |  |  |  |  |
| ***GCK*** (NM_000162.5) | | | | | | | | | | |
| 118 | exon 6 | c.589A>G | p.M197V | het | de novo | preterm | 4.81 | 1 | none | DZX |
| 119 | exon 10 | c.1363_1364insCGG | p.V455delinsAV | het | de novo | term | 4.5 | 2 | seizure | DZX |
| ***HNF4A*** (NM_175914) | | | | | | | | | | |
| 120 | exon 2 | c.187C＞T | p.R63W | het | de novo | term | 3.8 | 1 | none | DZX |
| 121 | exon 9 | c.1144G＞A | p.V382I | het | maternal | term | 3.8 | 159 | seizure | DZX |

het, heterozygous; ch, compound heterozygous; none, asymptomatic hyperglycemia; AGA, appropriate for gestational age; LGA, large for gestational age; SGA, small for gestational age; n.d., be not determined; DZX, diazoxide; OCT, octreotide; refuse, patients forwent therapy due to drug side effects or potential surgical complications; focal or diffuse, ^18^F-DOPA-PET-CT and surgical intervention were undergone to confirm.

**Supplementary Table S2.** Clinical characteristics of 79 patients with *ABCC8* and *KCNJ11* variants.

|  | *ABCC8* | *KCNJ11* | *P* value |
| --- | --- | --- | --- |
| Cases, n (%) | 69 (87.3) | 10 (12.7) |  |
| Gender M/F (%) | 56.5/43.5 | 30.0/70.0 | 0.218 |
| Macrosomia, n (%) | 35 (50.7) | 4 (40.0) | 0.768 |
| Birth weight, kg | 4.00 (1.80~5.50) | 3.45 (3.00~4.55) | 0.165 |
| Severe symptoms at onset, n (%) | 32 (46.4) | 6 (60.0) | 0.640 |
| Days at presentation, days | 2 (1-690) | 19.5 (1-143) | 0.807 |
| Serum insulin, mU/L | 13.29 (1.66-110.60) | 12.64 (3.97-30.51) | 0.988 |
| Serum C-peptide, nmol/L | 3.21 (0.60-8.29) | 3.04 (1.78-9.17) | 0.883 |
| Therapeutic options, n (%) |  |  | 1.000^a^ |
| Frequent feeding | 1 (1.4) | n.p. |  |
| Diazoxide treatment | 16 (23.2) | 2 (20.0) |  |
| Octreotide treatment | 28 (40.6) | 5 (50.0) |  |
| Surgery (Focal) | 8 (11.6) | n.p. |  |
| Surgery (Diffuse) | 4 (5.8) | 2 (20.0) |  |
| Abandon treatment | 12 (17.4) | 1 (10.0) |  |

M, male; F, female; n.p., no patient.

^a^We made comparison based on whether patients were responsive to non-surgical treatments, excluding patients who have given up treatment. The statistical methods used in other tables are consistent with those used in this table.

Counting variables represented as median (range) values. Categorical variables are represented as the number (n) and percentage (%) of the patients. Comparisons of counting variables were performed by the Mann–Whitney U test; categorical variables were performed by the Pearson's chi-squared test or Yates's correction for continuity.

**Supplementary Table S3.** Clinical characteristics of 69 patients with *ABCC8* variants.

|  | p.G111R | other *ABCC8* variants | *P* value |
| --- | --- | --- | --- |
| Cases, n (%) | 10 (14.5) | 59 (85.5) |  |
| Gender M/F (%) | 70.0/30.0 | 54.2/45.8 | 0.579 |
| Macrosomia, n (%) | 5 (50.0) | 30 (50.8) | 1.000 |
| Birth weight, kg | 3.55 (2.70~4.50) | 3.45(2.64~4.10) |  |
| Initial Symptoms, n (%) | 4 (40.0) | 28 (47.5) | 0.925 |
| Days at presentation, days | 1.5 (1-162) | 2.0 (1-690) | 0.270 |
| Serum insulin, mU/L | 24.42 (5.82-69.00) | 11.90 (1.66-110.60) | 0.110 |
| Serum C-peptide, nmol/L | 4.15 (1.63-7.47) | 2.96 (0.60-8.29) | 0.115 |
| Therapeutic options, n (%) |  |  | 0.445 |
| Frequent feeding | n.p. | 1 (1.7) |  |
| Diazoxide treatment | n.p. | 16 (27.1) |  |
| Octreotide treatment | 5 (50.0) | 23 (40.0) |  |
| Surgery (Focal) | 2 (20.0) | 6 (10.2) |  |
| Surgery (Diffuse) | 1 (10.0) | 3 (5.1) |  |
| Abandon treatment | 2 (20.0) | 10 (16.9) |  |

M, male; F, female; n.p., no patient.

Counting variables represented as median (range) values. Categorical variables are represented as the number (n) and percentage (%) of the patients. Comparisons of counting variables were performed by the Mann–Whitney U test, and categorical variables were performed by the Yates's correction for continuity.

**Supplementary Table S4 information:**


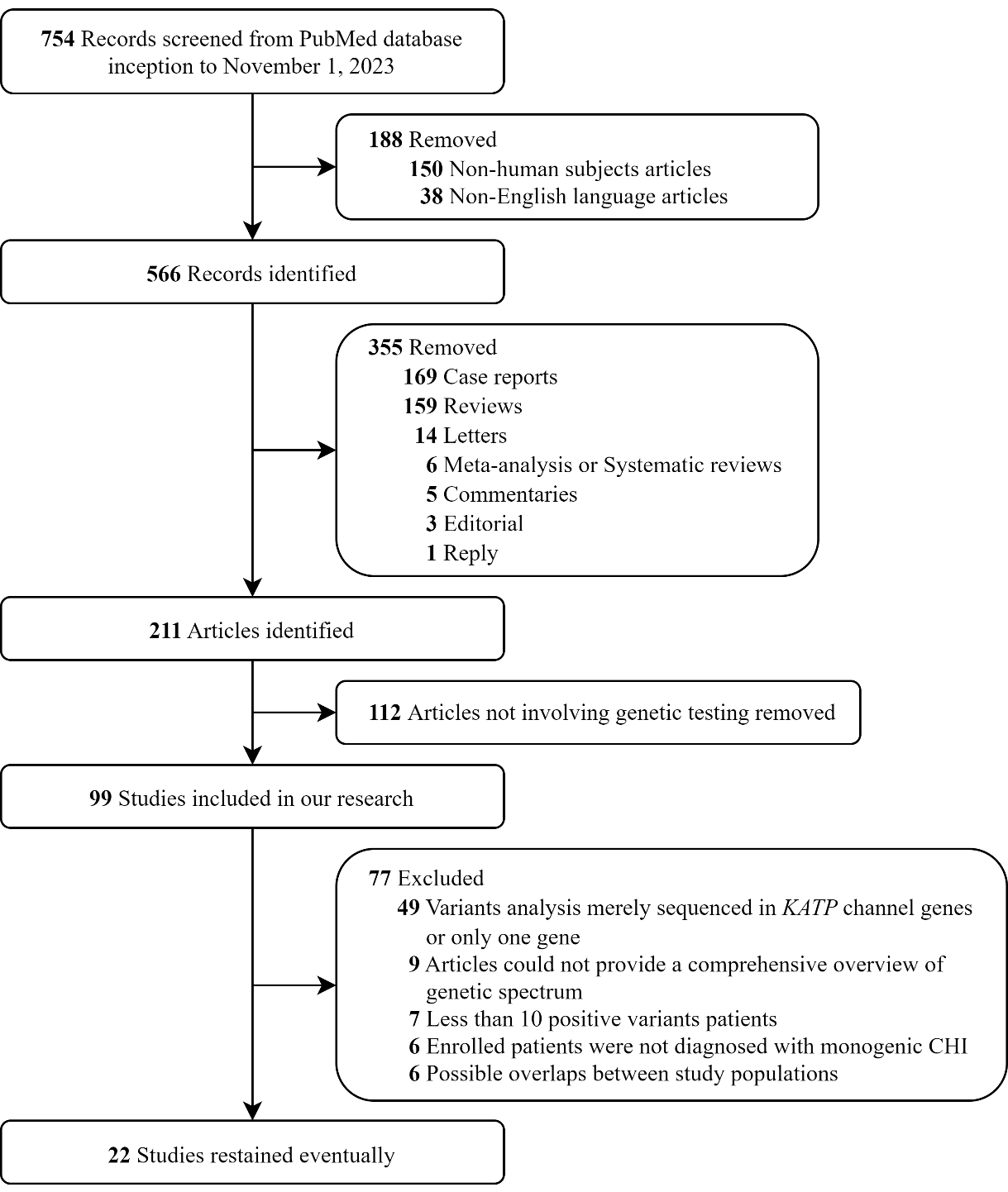
To investigate the genetic spectrum of monogenic CHI, we conducted a preliminary search from PubMed database (search with the term congenital hyperinsulinism[Title/Abstract]). The flow chart of the study selection process is detailed as follows. Articles should meet the following criteria: 1) a study with ten or more positive variant cases; 2) variants analysis sequenced in *KATP* channel gene and any one or more gene associated with monogenic CHI; 3) a study not involving a specific CHI group, such as diazoxide-unresponsive CHI, or assessments of drug efficacy and safety; 4) a study with the highest number of positive variant cases was selected when possibly overlaps between study populations.

**Supplementary Table S4.** Spectrum of positive variant patients with monogenic CHI from different regions.

| Region | Positive cases | Diazoxide-responsiveness^#^ | *ABCC8* | *KCNJ11* | *GLUD1* | *GCK* | *HADH* | *HNF1A* | *HNF4A* | *SLC16A1* | *UCP2* | Reference |
| --- | --- | --- | --- | --- | --- | --- | --- | --- | --- | --- | --- | --- |
| UK^a^ | 1253 | N.A. | 919 (72.8%) | 115 (9.1%) | 72 (5.7%) | 22 (1.7 %) | 67 (5.3%) | 8 (0.6%) | 48 (3.8%) | N.D. | N.D. | （1） |
| UK^b^ | 136 | 15 (13.8%) | 98 (72.0%) | 11 (8.1%) | 16 (11.8%)^*^ | - | 3 (2.2%) | - | 8 (5.9%) | N.D. | N.D. | (2) |
| USA | 328 | 23 (8.0%) | 261(79.5%) | 27 (8.2%) | 24 (7.2%) | 7 (2.1%) | 3 (0.9%) | 3 (0.9%) | 2 (0.6%) | - | 2 (0.6%) | (3) |
| UK^c^ | 35 | N.A. | 27 (77.1%) | 5 (14.2%) | 1(2.9%)^*^ | 1(2.9%) | N.D. | N.D. | 1(2.9%) | N.D. | N.D. | (4) |
| UK^d^ | 12 | 2 (22.2%) | 4 (33.3%) | 5 (41.7%) | 3 (25.0%) | - | - | - | - | - | - | (5) |
| Finland | 65 | 20 (37.7%) | 49 (75.4%) | 4 (6.2%) | 6 (9.2%) | 2 (3.1%) |  |  | 1 (1.5%) | 3 (4.6%) | - | (6) |
| Germany | 28 | N.A. | 18 (64.3%) | 5 (17.8%) | 2 (7.1%) | 3 (10.8%) | - | - | - | - | - | (7) |
| Turkey | 12 | 4 (40.0%) | 9 (75.0%) | 1 (8.3%) | 1 (8.3%)^*^ | N.D. | 1 (8.3%) | N.D. | - | N.D. | N.D. | (8) |
| Turkey | 18 | 1 (5.9%) | 14 (77.8%) | 3 (16.7%) | N.D. | N.D. | 1 (5.5%) | N.D. | - | N.D. | N.D. | (9) |
| Spain | 28 | 4 (19.0%) | 20 (71.4%) | 1 (3.6%) | 3 (10.7%) | 4 (14.3%) | - | - | - | N.D. | N.D. | (10) |
| Italy | 20 | 5 (41.7%) | 11 (55.0%) | 1 (5.0%) | 5 (25.0%) | 2 (10.0%) | - | N.D. | 1 (5.0%) | N.D. | N.D. | (11) |
| Italy | 16 | 6 (50.0%) | 12 (75.0%) | - | 1 (6.2%) | 2 (12.6%) | 1 (6.2%) | - | - | - | - | (12) |
| Brazil | 33 | N.A. | 15 (45.5%) | 6 (18.2%) | 9 (27.3%) | 3 (9.0%) | - | - | - | - | N.D. | (13) |
| Egypt | 21 | N.A. | 17 (81.0%) | 1 (4.8%) | 1 (4.8%) | 2 (9.4%) | - | - | - | - | N.D. | (14) |
| Iran^e^ | 23 | 11 (55.0%) | 17 (74.0%) | 3 (13.0%) | - | 3 (13.0%) | - | - | - | - | - | (15) |
| Oman^f^ | 25 | N.A. | 25(100.0%) | - | - | - | - | - | - | - | - | (16) |
| India^g^ | 27 | 3 (13.6%) | 22 (81.5%) | - | 3 (11.1%) | - | 2 (7.4%) | - | - | - | N.D. | (17) |
| South India | 10 | 2 (20.0%) | 10 (100.0%) | - | -^*^ | - | N.D. | N.D. | - | N.D. | N.D. | (18) |
| Japan | 24 | 2 (10.5%) | 18 (75.0%) | 1 (4.2%) | 5 (20.8%) | - | N.D. | N.D. | N.D. | N.D. | N.D. | (19) |
| Korea | 14 | 4 (28.6%) | 11 (78.6%) | 3 (21.4%) | N.D. | - | N.D. | N.D. | N.D. | N.D. | N.D. | (20) |
| South China | 21 | 12 (57.1%) | 16 (76.2%) | - | 5 (23.8%) | - | N.D. | N.D. | N.D. | N.D. | N.D. | (21) |
| China^h^ | 24 | N.A. | 14 (58.3%) | 4 (16.7%) | 1 (4.2%) | 1 (4.2%) | 2 (8.3%) | N.D. | 2 (8.3%) | N.D. | - | (22) |
| This study | 121 | 18 (22.8%) | 69 (56.9%) | 10 (8.3%) | 35 (28.9%) | 2 (2.5%) | 3 (1.7%) | - | 2 (1.7%) | - | - |  |

N.A. Not available; N.D. Not done

^#^Diazoxide-responsiveness in patients with *KATP* channel mutations.

^*^The *GLUD1* gene was sequenced in patients with hyperinsulinism and hyperammonemia.

^a^The remaining genotypes comprise *PMM2* variants (n=10, 0.8% ) and *CACNA1D* variants (n=2, 0.2%), excluding *INSR*, *KMT2D*, *KDM6A*, and *TRMT10A* (n=27).

^b^Patients came from the Great Ormond Street Children’s Hospital, which is a national and international center for CHI.

^c^Patients came from northern UK.

^d^Patients came from Royal Hospital for Children, Glasgow, UK.

^e^Twenty-four positive variant cases totally, with one patient with *KMT2D* variant.

^f^This study did not specify the candidate genes for testing other than *ABCC8*, *KCNJ11*, *GCK* and *HNF4A*.

^g^Twenty-eight positive variant cases totally, with one patient with *KMD6A* variant.

^h^Patients came from a children’s national medical center (Shanghai, China).

**Supplementary Table S5.** Summary of patients with *ABCC8* variant (p.G111R) reported previously.

| No. of Cases | Region | Gender | Days at presentation | Symptom | Treatment | Nucleotide | Amino acid | Genetic status | Parental origin | Reference |
| --- | --- | --- | --- | --- | --- | --- | --- | --- | --- | --- |
| 1 | China | male | 1 | asymptomatic hypoglycemia | octreotide | c.331G>A | p.G111R | HET | paternal | this study |
| 2 | China | male | 2 | seizure | surgery (focal) | c.331G>A | p.G111R | HET | paternal | this study |
| 3 | China | male | 2 | lethargy | abandon^#^ | c.331G>A | p.G111R | HET | paternal | this study |
| 4 | China | male | 1 | asymptomatic hypoglycemia | abandon^#^ | c.331G>A | p.G111R | HET | paternal | this study |
| 5 | China | male | 124 | unconscious | octreotide | c.331G>A | p.G111R | HET | paternal | this study |
| 6 | China | female | 2 | seizure | surgery (focal) | c.331G>A | p.G111R | HET | paternal | this study |
| 7 | China | female | 162 | seizure | octreotide | c.331G>A | p.G111R | HET | paternal | this study |
| 8 | China | female | 1 | asymptomatic hypoglycemia | octreotide | c.331G>A | p.G111R | CH | paternal | this study |
|  |  |  |  |  |  | c.4612C>T | p.R1538* |  | maternal |  |
| 9 | China | male | 1 | asymptomatic hypoglycemia | octreotide | c.331G>A | p.G111R | CH | paternal | this study |
|  |  |  |  |  |  | c.1792C>T | p.R598* |  | maternal |  |
| 10 | China | male | 1 | asymptomatic hypoglycemia | surgery (diffuse) | c.331G>A | p.G111R | CH | paternal | this study |
|  |  |  |  |  |  | c.1817+2T＞C | p.? |  | maternal |  |
| 11 | China | N.A. | 2 | N.A. | surgery (focal) | c.331G>A | p.G111R | HET | N.A. | (23) |
| 12 | Japan | male | 1 | N.A. | surgery (focal) | c.331G>A | p.G111R | HET | paternal | (19) |
| 13 | Northern India | N.A. | 8 | N.A. | octreotide | c.331G>A | p.G111R | CH | N.A. | (17) |
|  |  |  |  |  |  | c.3979G>T | p.E1327* |  | N.A. |  |
| 14 | Northern India | N.A. | 1 | N.A. | octreotide | c.221G>A | p.R74Q | CH | N.A. | (17) |
|  |  |  |  |  |  | c.331G>A | p.G111R |  | N.A. |  |
| 15 | Northern India | N.A. | 1 | N.A. | octreotide | c.331G>A | p.G111R | CH | N.A. | (17) |
|  |  |  |  |  |  | c.4411G>A | p.D1471N |  | N.A. |  |
| 16 | Northern India | N.A. | 1 | N.A. | octreotide | c.331G>A | p.G111R | HOM | biparental | (17) |
| 17 | Northern India | N.A. | 2 | N.A. | octreotide | c.331G>A | p.G111R | HOM | biparental | (17) |
| 18 | Northern India | N.A. | 6 | N.A. | diazoxide | c.331G>A | p.G111R | HET | paternal | (17) |
| 19 | South India | female | 1 | N.A. | surgery (diffuse) | c.331G>A | p.G111R | HOM | biparental | (24) |
| 20 | Netherlands | female | 1 | lethargy | surgery (focal) | c.331G>A | p.G111R | HET | paternal | (25) |
| 21 | UK | N.A. | 3 | N.A | surgery (focal) | c.331G>A | p.G111R | HET | paternal | (2) |
| 22 | UK | N.A. | 1 | N.A. | surgery (diffuse) | c.331G>A | p.G111R | HOM | biparental | (2) |
| 23 | UK | N.A. | 1 | N.A. | surgery (diffuse) | c.331G>A | p.G111R | HOM | biparental | (2) |
| 24 | UK | N.A. | 1 | N.A. | surgery (diffuse) | c.331G>A | p.G111R | HOM | biparental | (2) |
| 25 | Brazil | N.A. | N.A. | N.A. | N.A. | c.331G>A | p.G111R | N.A. | N.A. | (13) |

LGA, Large for gestational age; AGA, Appropriate for gestational age; N.A. Not available; HET, Heterozygote; CH, Compound heterozygote; HOM, Homozygote.

^#^These two patients were diazoxide-unresponsive and then discontinued treatments.


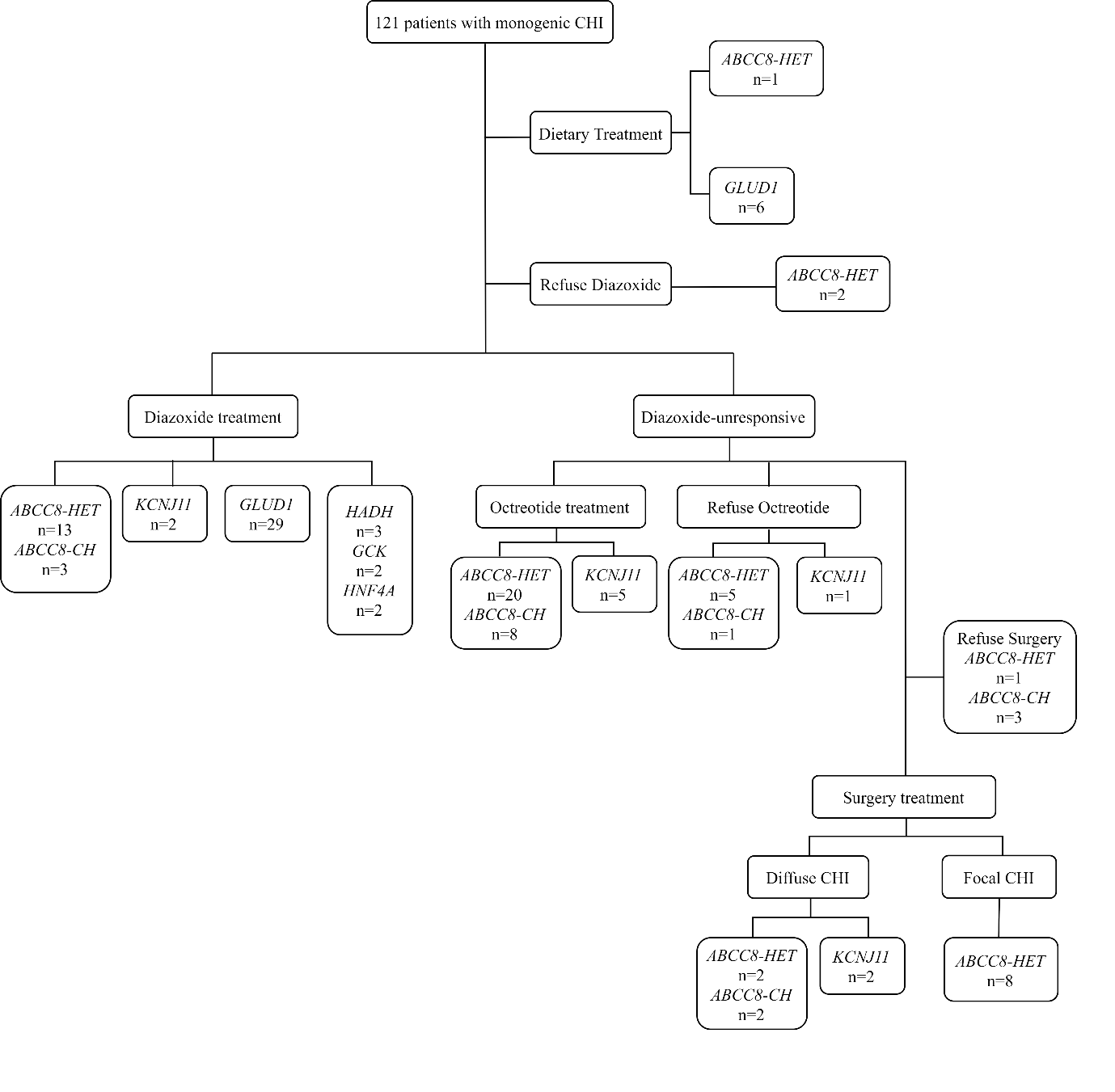


**Supplementary Figure S1.** The flowchart of therapeutic options of 121 patients based on genotypes. The number of patients of each genotype is represented as number (n). HET, Heterozygote; CH, Compound heterozygote.

**Supplementary References**

1. Hopkins JJ, Childs AJ, Houghton JAL, et al. Hyperinsulinemic hypoglycemia diagnosed in childhood can be monogenic. *J Clin Endocrinol Metab*. 2023;108(3):680-687.

2. Kapoor RR, Flanagan SE, Arya VB, Shield JP, Ellard S, Hussain K. Clinical and molecular characterisation of 300 patients with congenital hyperinsulinism. *Eur J Endocrinol*. 2013;168(4):557-564.

3. Snider KE, Becker S, Boyajian L, et al. Genotype and Phenotype Correlations in 417 Children With Congenital Hyperinsulinism. *J Clin Endocrinol Metab*. 2013;98(2):E355-E363.

4. Banerjee I, Skae M, Flanagan SE, et al. The contribution of rapid KATP channel gene mutation analysis to the clinical management of children with congenital hyperinsulinism. *Eur J Endocrinol*. 2011;164(5):733-740.

5. El Tonbary K, Robinson P, Banerjee I, Shaikh MG. Congenital hyperinsulinism: management and outcome, a single tertiary centre experience. *Eur J Pediatr*. 2020;179(6):947-952.

6. Männistö JME, Maria M, Raivo J, Kuulasmaa T, Otonkoski T. Clinical and genetic characterization of 153 patients with persistent or transient congenital hyperinsulinism. *J Clin Endocrinol Metab*. 2020;105(4):e1686-e1694.

7. Ludwig A, Enke S, Heindorf J, Empting S, Meissner T, Mohnike K. Formal Neurocognitive Testing in 60 Patients with Congenital Hyperinsulinism. *Horm Res Paediatr*. 2018;89(1):1-6.

8. Güven A, Cebeci AN, Ellard S, Flanagan SE. Clinical and Genetic Characteristics, Management and Long-Term Follow-Up of Turkish Patients with Congenital Hyperinsulinism. *J Clin Res Pediatr Endocrinol*. 2016;8(2):197-204.

9. Demirbilek H, Arya VB, Ozbek MN, et al. Clinical characteristics and phenotype–genotype analysis in turkish patients with congenital hyperinsulinism; predominance of recessive KATP channel mutations. *Eur J Endocrinol*. 2014;170(6):885-892.

10. Martínez R, Fernández-Ramos C, Vela A, et al. Clinical and genetic characterization of congenital hyperinsulinism in spain. *Eur J Endocrinol*. 2016;174(6):717-726.

11. Faletra F, Athanasakis E, Morgan A, et al. Congenital hyperinsulinism: Clinical and molecular analysis of a large Italian cohort. *Gene*. 2013;521(1):160-165.

12. Casertano A, De Matteis A, Mozzillo E, et al. Diagnosis of congenital Hyperinsulinism can occur not only in infancy but also in later age: a new flow chart from a single center experience. *Ital J Pediatr*. 2020;46(1):131.

13. Del Roio Liberatore R, Ramos PM, Guerra G, Manna TD, Silva IN, Martinelli CE. Clinical and molecular data from 61 Brazilian cases of Congenital Hyperinsulinemic Hypoglycemia. *Diabetol Metab Syndr*. 2015;7(1):5.

14. Laimon W, Aboelenin HM, El Tantawi NT. Clinical characteristics, outcome, and predictors of neurological sequelae of persistent congenital hyperinsulinism: A single tertiary center experience. *Pediatr Diabetes*. 2021;22(3):388-399.

15. Razzaghy-Azar M, Saeedi S, Dayani SB, et al. Investigating Genetic Mutations in a Large Cohort of Iranian Patients with Congenital Hyperinsulinism. *J Clin Res Pediatr Endocrinol*. 2022;14(1):87-95.

16. Al-Badi MK, Al-Azkawi HS, Al-Yahyaei MS, Mula-Abed WA, Al-Senani AM. Clinical characteristics and phenotype-genotype review of 25 Omani children with congenital hyperinsulinism in infancy: A one-decade single-center experience. *Saudi Med J*. 2019;40(7):669-674.

17. Sharma R, Roy K, Satapathy AK, et al. Molecular Characterization and Management of Congenital Hyperinsulinism: A Tertiary Centre Experience. *Indian Pediatr*. 2022;59(2):105-109.

18. Jahnavi S, Poovazhagi V, Kanthimathi S, et al. Novel *ABCC8* ( *SUR1* ) Gene Mutations in Asian Indian Children with Congenital Hyperinsulinemic Hypoglycemia: *ABCC8* Mutations in Indian CHI Children. *Ann Hum Genet*. 2014;78(5):311-319.

19. Yorifuji T, Kawakita R, Nagai S, et al. Molecular and Clinical Analysis of Japanese Patients with Persistent Congenital Hyperinsulinism: Predominance of Paternally Inherited Monoallelic Mutations in the K _ATP_ Channel Genes. *J Clin Endocrinol Metab*. 2011;96(1):E141-E145.

20. Park SE, Flanagan SE, Hussain K, Ellard S, Shin CH, Yang SW. Characterization of ABCC8 and KCNJ11 gene mutations and phenotypes in Korean patients with congenital hyperinsulinism. *Eur J Endocrinol*. 2011;164(6):919-926.

21. Xu A, Cheng J, Sheng H, et al. Clinical management and gene mutation analysis of children with congenital hyperinsulinism in south China. *J Clin Res Pediatr Endocrinol*. 2019;11(4):400-409.

22. Fan Z, Ni J, Yang L, et al. Uncovering the molecular pathogenesis of congenital hyperinsulinism by panel gene sequencing in 32 Chinese patients. *Mol Genet Genomic Med*. 2015;3(6):526-536.

23. Ni J, Ge J, Zhang M, et al. Genotype and phenotype analysis of a cohort of patients with congenital hyperinsulinism based on DOPA-PET CT scanning. *Eur J Pediatr*. 2019;178(8):1161-1169. doi:10.1007/s00431-019-03408-6

24. Jahnavi S, Poovazhagi V, Kanthimathi S, et al. Novel *ABCC8* ( *SUR1* ) Gene Mutations in Asian Indian Children with Congenital Hyperinsulinemic Hypoglycemia: *ABCC8* Mutations in Indian CHI Children. *Ann Hum Genet*. 2014;78(5):311-319.

25. De Vroede M, Bax NMA, Brusgaard K, Dunne MJ, Groenendaal F. Laparoscopic Diagnosis and Cure of Hyperinsulinism in Two Cases of Focal Adenomatous Hyperplasia in Infancy. *Pediatrics*. 2004;114(4):e520-e522.
